# Myosin Va Participates in Acrosomal Formation and Nuclear Morphogenesis during Spermatogenesis of Chinese Mitten Crab *Eriocheir sinensi*s

**DOI:** 10.1371/journal.pone.0012738

**Published:** 2010-09-14

**Authors:** Xiao Sun, Ying He, Lin Hou, Wan-Xi Yang

**Affiliations:** 1 The Sperm Laboratory, College of Life Sciences, Zhejiang University, Hangzhou, China; 2 College of Life Sciences, Liaoning Normal University, Dalian, China; Institute of Zoology, Chinese Academy of Sciences, China

## Abstract

**Background:**

The Chinese mitten crab *Eriocheir sinensis* belongs to the Class Crustacea, Decapoda, Brachyura. The spermatozoon of this species is of aflagellated type, it has a spherical acrosome surrounded by the cup-shaped nucleus, which are unique to brachyurans. For the past several decades, studies on the spermatogenesis of the mitten crab mainly focus on the morphology. Compared with the extensive study of molecular mechanism of spermatogenesis in mammals, relatively less information is available in crustacean species. Myosin Va, a member of Class V myosin, has been implicated in acrosome biogenesis and vesicle transport during spermatogenesis in mammals. In the present study we demonstrate the expression and cellular localization of myosin Va during spermatogenesis in *E. sinensis*.

**Methodology/Principal Findings:**

Western blot demonstrated that myosin Va is expressed during spermatogenesis. Immunocytochemical and ultrastructural analyses showed that myosin Va mainly localizes in the cytoplasm in spermatocytes. At the early stage of spermiogenesis, myosin Va binds to the endoplasmic reticulum vesicle (EV) and proacrosomal granule (PG). Subsequently, myosin Va localizes within the proacrosomal vesicle (PV) formed by PG and EV fusion and locates in the membrane complex (MC) at the mid spermatid stage. At the late spermatid stage, myosin Va is associated with the shaping nucleus and mitochondria. In mature spermatozoon, myosin Va predominates in acrosomal tubule (AT) and nucleus.

**Conclusions/Significance:**

Our study demonstrates that myosin Va may be involved in acrosome biogenesis and nuclear morphogenesis during spermatogenesis in *E. sinensis*. Considering the distribution and molecular characteristics of myosin Va, we also propose a hypothesis of AT formation in this species. It is the first time to uncover the role of myosin Va in crustacean spermatogenesis.

## Introduction

Spermatogenesis refers to the process which diploid spermatogonia develop into haploid mature spermatozoa. It can be divided into three phases: the mitotic phase, meiotic phase, and spermiogenesis phase. At the mitotic phase, the spermatogonia develop into primary spermatocytes through mitosis. During the meiotic phase, primary spermatocytes change into secondary spermatocytes via meiosis. At the spermiogenesis phase, spermatids differentiate into well shaped spermatozoa through organelle reorganization and new structure formation [1], [2]. Three major events occur during spermiogenesis in mammals, i.e., nuclear condensation, acrosomal shaping, and mid-piece formation.

**Figure 1:**
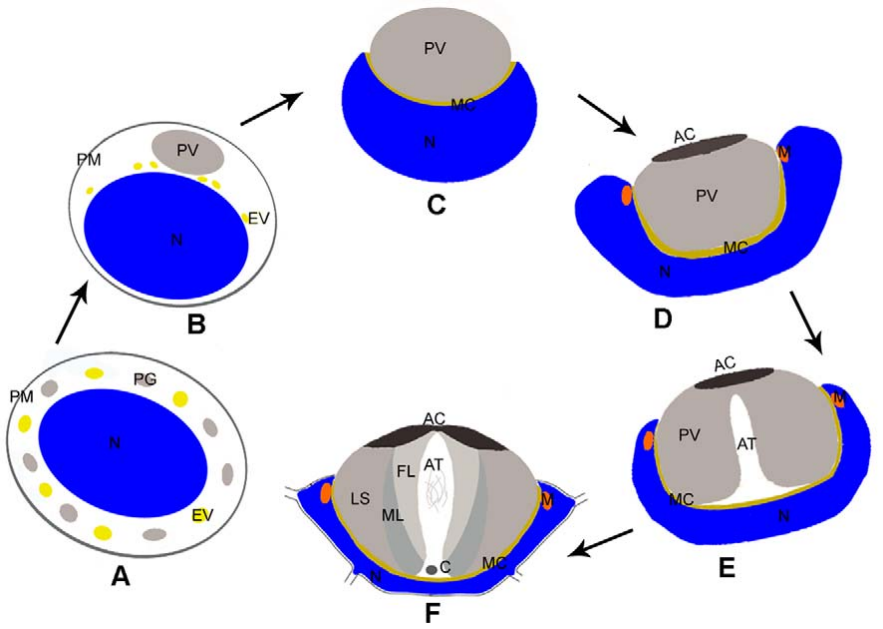
A model of spermiogenesis in Chinese mitten crab *E. sinensis*. (A) At early stage proacrosomal granule (PG) and the endoplasmic reticulum vesicle (EV) distribute in the spermatid cytoplasm around the nucleus (N). PM indicates the plasma membrane. (B–C) At mid stage, proacrosomal granule (PG) and the endoplasmic reticulum vesicle (EV) aggregate into proacrosomal vesicle (PV) (B), subsequently, the nucleus (N) initiates to wrap up proacrosomal vesicle (PV) and the membrane complex (MC) emerges between proacrosomal vesicle (PV) and the nucleus (N) (C). The spermatid discards most of the cytoplasm (C). (D–F) At late stage, the proacrosomal vesicle (PV) invaginates to form the acrosomal tubule (AT) (D, E). The mature spermatozoon consists of acrosome with apical cap (AC), acrosomal tubule (AT) and three layers (fibrous layer FL, middle layer ML and lamellar structures LS), centriole (C) at the base of acrosomal tubule (AT), the nuclear cup with several radial arms (RA) and mitochondria (M) (F).

However, not all the sperm share the same features during spermiogenesis. In most crustaceans, the sperm has a characteristic acrosome complex and has no flagellum [3]–[6]. Chinese mitten crab *Eriocheir sinensis* (Crustacea, Decapoda) is a commercial aquatic species widely bred in China. It has been a gastronomic favorite of consumers for centuries. In recent two decades, this species has been used as a model for studying the crustacean spermatogenesis. Studies on the mechanism of spermatogenesis of this species are helpful to the culture and management of this crab. Spermiogenesis in the Chinese mitten crab *E. sinensis* has been divided into three stages, the early stage, mid stage and late stage (Figure 1) [3]. At the early stage, the endoplasmic reticulum vesicle (EV) generates dense proacrosomal granule (PG), which distribute in the spermatid cytoplasm (Figure 1A). At the mid stage, the proacrosomal granule (PG) and part of the endoplasmic reticulum vesicle (EV) aggregate into one vesicle designated as proacrosomal vesicle (PV) adjacent to the nucleus (Figure 1B). Later, the nucleus initiates to wrap up PV and a thin layer of membrane complex (MC) is sandwiched between the PV and the nucleus, and then the spermatid discards most of the cytoplasm (Figure 1C). During the late stage, the PV invaginates to form the acrosomal tubule (AT) and some mitochondria are surrounded by the nuclear membrane at subapical region of the spermatid (Figure 1D, E); finally, the nucleus becomes cup-shaped with several radial arms (RA) surrounding the acrosome (Figure 1F). In mature spermatozoon, a nuclear cup surrounds the acrosome, the latter is composed of an apical cap (AC), three layers (fibrous layer FL, middle layer ML and lamellar structures LS) and the acrosomal tubule (AT) (Figure 1F) [7]–[9].

Comparing to the mammalian spermatozoon with the acrosome covered most of the elongated nucleus, the Chinese mitten crab *E. sinensis* spermatozoon has a characteristic organization with a cup-shaped nucleus surrounded the acrosomal complex. Motor proteins play important roles during acrosome biogenesis and nuclear morphogenesis [9]–[13]. KIFC1, a microtubule-dependent molecular motor, has been shown in acrosomal formation during spermiogenesis in the Chinese mitten crab *E. sinensis* in our lab [9]. Class V myosin (myosin V), actin-based molecular motor, has a head domain with actin-binding site, a neck domain, and a tail domain responsible for cargo binding and is involved in a diverse range of cellular functions [14], [15]. Myosin V transports multiple cargos along actin filaments, including membrane-bound organelles and vesicles, such as ER vesicles [16], [17], mitochondria [18], [19], melanosomes [20], secretory granules in neurons [21], [22], endosomes and AMPA receptors [23], [24], GLUT4 vesicles [25], neurofilaments [26], mRNA and mRNP complex [27]–[32]. It is also associated with microtubules and kinesins [33]–[38]. Myosin V has been demonstrated to be involved in spermatogenesis [11], [12], [39]. It has a role in shaping and stabilizing the actin-based investment cones during *Drosophila* spermiogenesis [12]. Myosin Va, a member of myosin V, has been shown to transport the Golgi originated proacrosomal vesicles to the acroplaxome, then facilitate these vesicles coalescing into the acrosome [40]. It has also been suggested that myosin Va is involved in anchoring the acrosome to the acroplaxome and mobilizing vesicles along F-actin during the intramanchette transport [10]. Given the special pattern of spermatogenesis in *E. sinensis* and molecular characteristic of myosin Va, we determine to study the function of myosin Va during spermatogenesis in Chinese mitten crab *E. sinensis*.

In the present study, we report the expression of myosin Va during spermatogenesis in *E. sinensis*. We demonstrate that myosin Va is associated with EV and PG at an early spermatid stage and is localized in PV and MC at the mid spermatid stage. At the late spermatid stage, myosin Va bounds to mitochondria and distributes within the differentiating nucleus and AT. It suggests a role of myosin Va in acrosome biogenesis and nuclear shaping. We also suppose a model about the AT formation. Our findings reveal the first time that myosin Va may function in acrosome biogenesis and nuclear morphogenesis during crustacean spermatogenesis.

## Materials and Methods

### Animals

Seventy adult males of *E. sinensis* (Crustacea, Decapoda, Brachyura) were obtained from a local fishery market in Hangzhou, China. The specimens were maintained in aerated tanks for two weeks. Six adult male ICR mice were purchased from the Zhejiang University Laboratory Animal Center, and animal usage was also approved by the center. The permit number is SCXK 2007-0029. All procedures were carried out in accordance with the laws and polices by this animal center. For use of Chinese mitten crab, no approval is needed in China.

### Antibodies

Anti-Myosin Va (LF-18) was purchased from Sigma (St. Louis, Mo., USA). Rabbit Anti-β-actin was purchased from BIOS (China). FITC Conjugated Monoclonal Anti-actin was purchased from Sigma (St. Louis, Mo., USA). HRP Conjugated Goat anti-Rabbit IgG was purchased from Immunology Consultants Laboratory, Inc. Texas. Red-conjugated Affinipure Goat Anti-Rabbit IgG was purchased from Protein Tech Group, Inc.

### Western Blot Analysis

Testis from adult *E. sinensis* and brain from ICR mice were homogenized in RAPI Lysis Buffer (50 mM Tris–HCl pH 7.4, 150 mM NaCl, 1% NP-40, 0.1% SDS) containing protease inhibitors and centrifuged at 14,000 rpm. The supernatant were ultrafiltrated by Centricon Centrifugal Filter Unit YM-30 (Millipore). After that the protein concentration was determined by the Coomassie brilliant blue method. Samples containing equal amounts of protein were separated on 8% gels (SDS-PAGE) and electrophoretically transferred to the PVDF membrane (Bio-Rad). Anti-Myosin Va antibody was used at a dilution of 1∶200. The membrane were incubated with HRP-conjugated goat anti-rabbit IgG secondary antibody diluted 1∶1000. Immunoblots were developed using Pierce ECL Western Blotting Substrate (Thermo).

### Immunofluorescence

Testes were obtained from adult male *E. sinensis* and were fixed overnight in 4% paraformaldehyde (PFA) in phosphate buffered saline (0.1 M PBS, PH 7.4). The tissues were rinsed three times in PBS and were incubated overnight in 0.5 M sucrose in PBS, then were embedded in Tissue-Tek® O.C.T. Compound, and cut into 5-µm frozen sections. The sections were washed with PBS for 15 min at room temperature, then blocked in 6% BSA in TBST (20 mM Tris, pH 7.5, 154 mM NaCl, 2 mM EGTA, 2 mM MgCl2, 0.1% Triton X-100) for 1 hour. Testis sections were incubated with anti-myosin Va antibodies (1∶100 dilution) in blocking buffer (2% BSA, 0.1% azide in TBST) for 1 h, then rinsed 3 times in TBST. The primary antibody was detected with Goat anti-rabbit IgG conjugated to Texas Red (1∶200 dilution, Jackson Immuno Research Laboratories, West Grove, PA., USA). β-Actin was detected by monoclonal anti-β-actin conjugated to FITC (1∶100 dilution, Sigma, St. Louis, MO., USA) and nuclei were stained with DAPI contained in the mounting medium (Vectashield, Vector Laboratories). The intracellular localization of these proteins was observed with a LSM 510 fluorescence microscope fit with appropriate filters and images captured with an Orca II CCD camera (Hamamatsu, Bridgewater, NJ). For controls, the primary antibody was omitted (data not shown).

### Colloidal Gold Labeling and Electron Microscopy

Testes were removed from *E. sinensis* to a separate dish, fixed in 4% paraformaldehyde and 0.1% glutaraldehyde, rinsed 3 times in 0.1 M sodium phosphate buffer, pH 7.4, and one time in distilled water. Samples were then dehydrated with increasing ethanol concentrations and embedded in LR White (Ted Pella, Inc., Redding, CA) overnight, changed to fresh media, and transferred to gelatin capsules and polymerized at 50°C for 24 h. Samples were sectioned on a Reichert ultramicrotome and collected on nickel grids. Grids were incubated with PBS with 50 mM Glycine, pH 7.4 for 20 minutes to inactivate aldehyde. Grids were then blocked in 5% egg albumin for 2 h and then incubated overnight with anti-myosin Va antibodies (diluted 1∶50 in 0.05 M Tris, pH 7.4, 5% egg albumin) at 4°C. The grids were then incubated with goat anti-rabbit IgG conjugated to 25 nm gold (Amersham Pharmacia Biotech, Piscataway, N.J., USA) diluted 1∶10 in 0.02 M Tris, pH 8.2, 5% egg albumin at room temperature. The grids were then washed twice with sodium phosphate buffer, rinsed twice in distilled water, air dried for at least 30 min, and counterstained with uranyl acetate and lead citrate. The primary antibody was omitted in control sections.

## Results

### Identification of Myosin Va in the testis of *E. sinensis*

**Figure 2:**
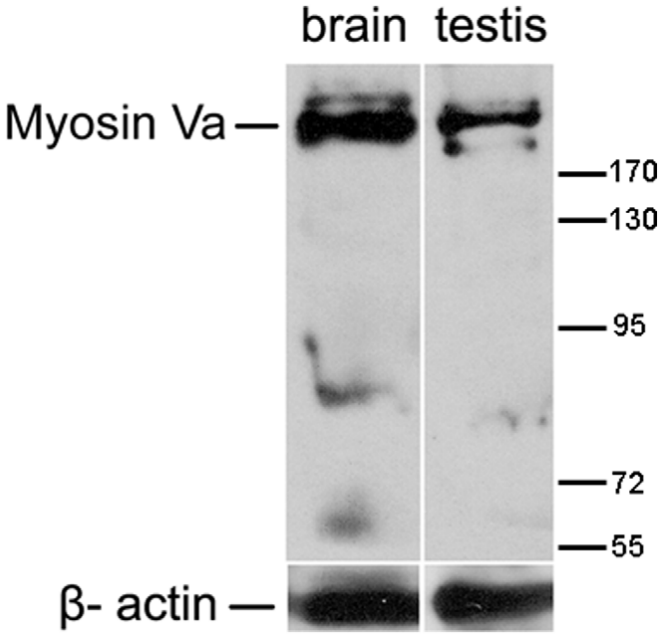
Myosin Va is expressed in the testis of *E. sinensis*. Immunoblots of myosin Va in *E. sinensis* testis and brain extracts of ICR male mice probed with the anti-myosin Va polyclonal antibody. Brain extracts of mice known to contain myosin Va serve as positive control. A similar band was observed in extracts of *E. sinensis* testis. β-actin was used as a loading control. The molecular weight marker is shown at right.

We performed western blot to determine whether myosin Va protein was expressed in the testis. The whole protein extraction of testis in *E. sinensis* and the brain extraction of ICR male mice known to contain neural vesicles rich in myosin Va were ultrafiltrated. Then, the concentrated solution was probed with an anti-myosin Va polyclonal antibody. In the positive control, the antibody recognized a band about 215 kDa expected for myosin Va in the brain lysate of mice, a similar immunoreactive band was indentified in testicular lysate of *E. sinensis* (Figure 2). The observed weak band maybe attributed to the products of alternatively spliced variants of myosin Va or the antibody cross-reacted with other members of class V myosins. Western blot analysis demonstrated that myosin Va is expressed in the testis of *E. sinensis*.

### Myosin Va localizes in the cytoplasm at spermatocytes

**Figure 3:**
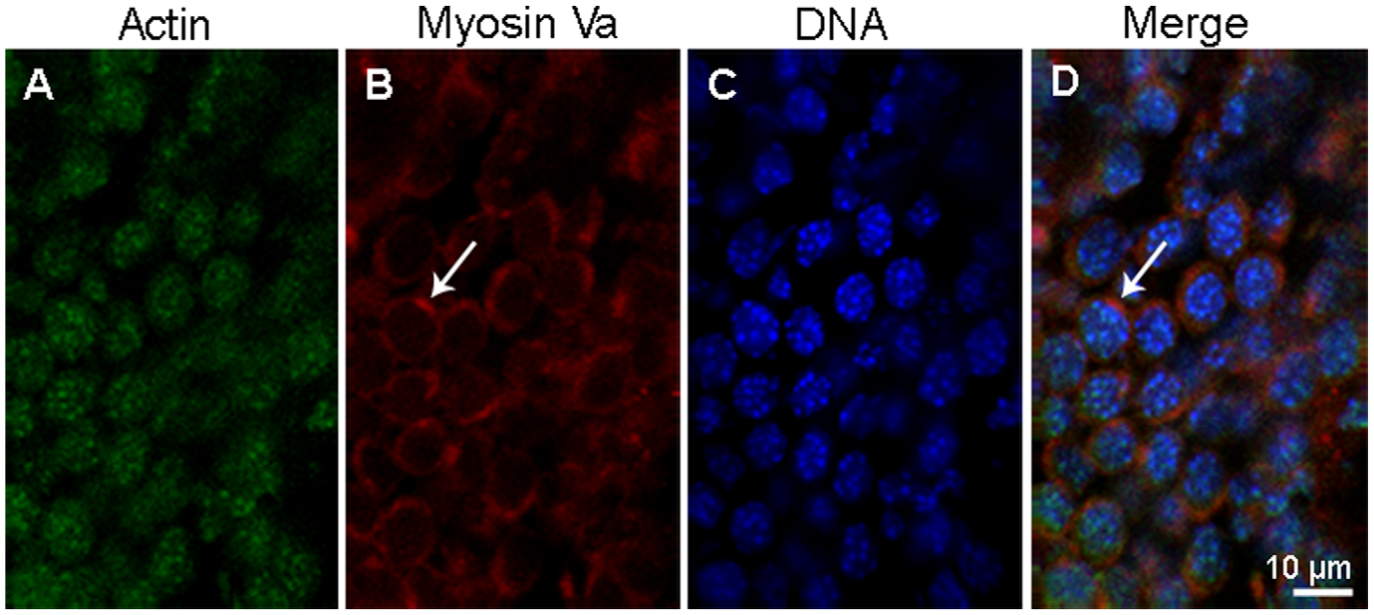
Immunofluorescent localization of myosin Va in spermatocytes of *E. sinensis*. Actin (green) is visualized with FITC conjugated monoclonal anti-actin, myosin Va (red), and DNA with DAPI (blue). Myosin Va mainly localizes in the cytoplasm (*arrows* in B, D) in spermatocytes; actin mainly localizes in the nucleus, while the cytoplasm presents weak diffuse staining. The chromatin granules dispersed in the nucleus can be seen (C).

Immunofluorescent localization results demonstrated that myosin Va distributes in the cytoplasm (Figure 3B, D). Actin shows strong punctuated staining within nucleus and weak diffuse staining in the cytoplasm (Figure 3A, C).

### Myosin Va is associated with the membrane of PG and EV at the early spermatid stage

**Figure 4:**
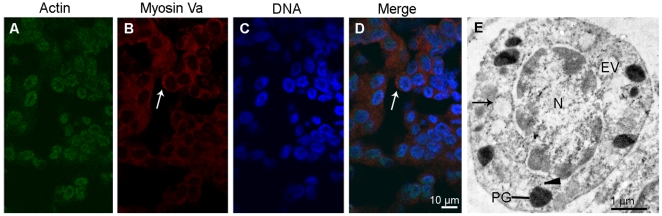
The localization of myosin Va at the early stage during *E. sinensis* spermiogenesis. (A–D) Immunofluorescent localization of myosin Va in early spermatids. The cytoplasm presents myosin Va staining (*arrows* in B, D), the chromatin lumps attach to the nuclear membrane (C). (e) Ultrastructural localization of myosin Va. Myosin Va binds to the endoplasmic reticulum vesicle (EV) membrane (*arrow*) and proacrosomal granule (PG) (*arrowhead*). The proacrosomal granule (PG) is characterized by high density while the endoplasmic reticulum vesicle (EV) has low density.

A pronounced feature of early spermatid is the formation of dense granule called PG, which originates from EV. Myosin Va staining is present in the cytoplasm, while the actin staining is prominent in the nucleus (Figure 4A–D). Myosin Va-decorated PG and EV can be observed in the cytoplasm (Figure 4E), which may suggest a role of myosin Va in PG formation and transportation.

### Myosin Va is principally localized in PV and MC at the mid spermatid stage

**Figure 5:**
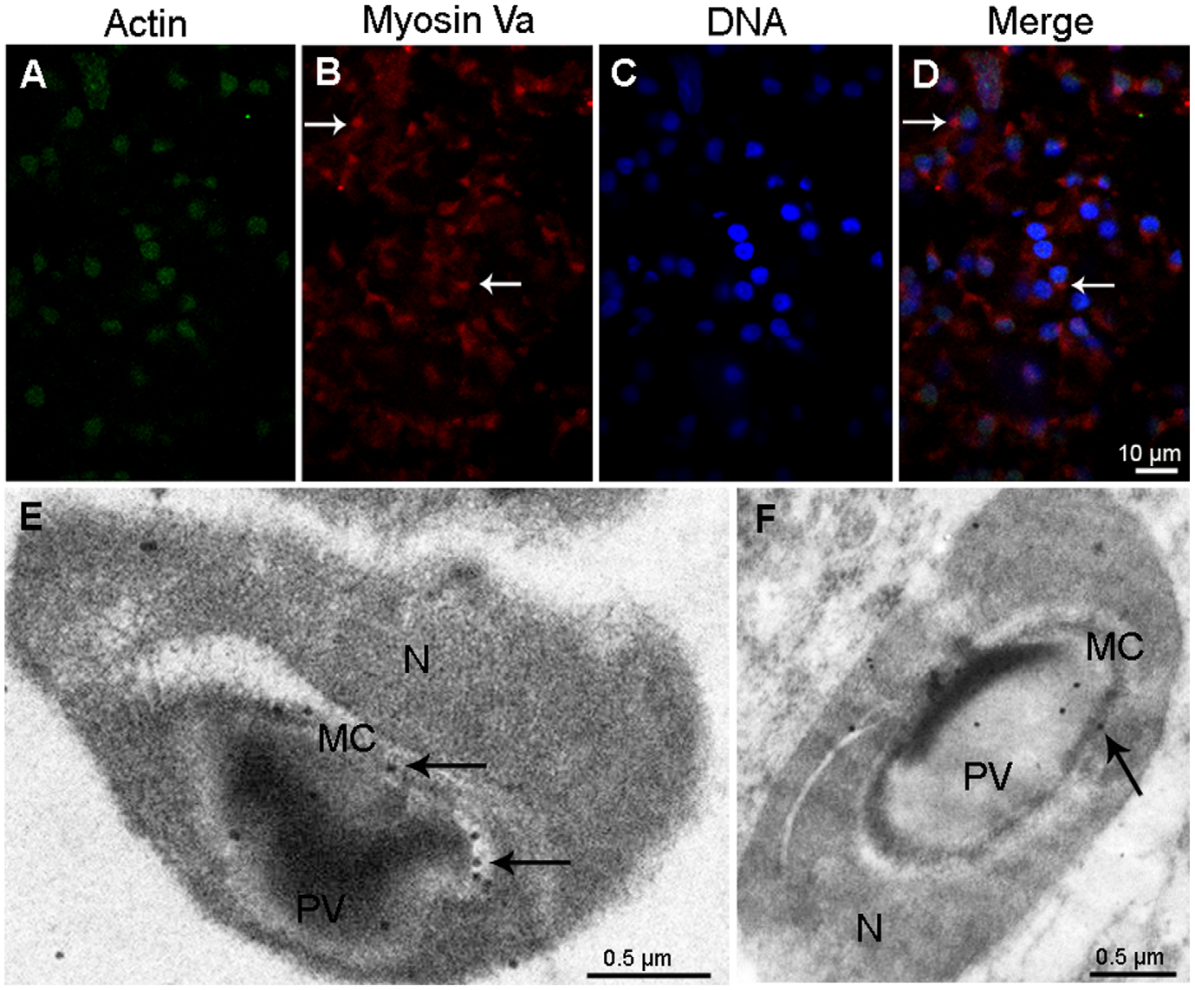
Localization of myosin Va at the mid stage during *E. sinensis* spermiogenesis. (A–D) Immunofluorescent localization of myosin Va in early mid stage. Myosin Va staining can be seen at the proacrosomal vesicle (PV) (*arrows* in B, D). Scale bar = 10 µm. (E–F) Immunogold labeling of myosin Va is present in the membrane complex (MC) and the proacrosomal vesicle (PV) membrane at late mid stage (*arrows* in E, F). E shows longitudinal section and F shows cross section of mid stage spermatid.

At the early mid spermatid stage, PG and EV accumulate and then fuse to form a large PV close to the nucleus, myosin Va immunoreactive sites can be seen at the PV in the spermatid (Figure 5A–D). Immunogold labeling of myosin Va are visualized along the PV membrane and MC, with a small quantity within the PV at the late mid spermatid stage (Figure 5E, F).

### Myosin Va localizes in the nucleus, mitochondria, MC and AT at the late spermatid stage

**Figure 6:**
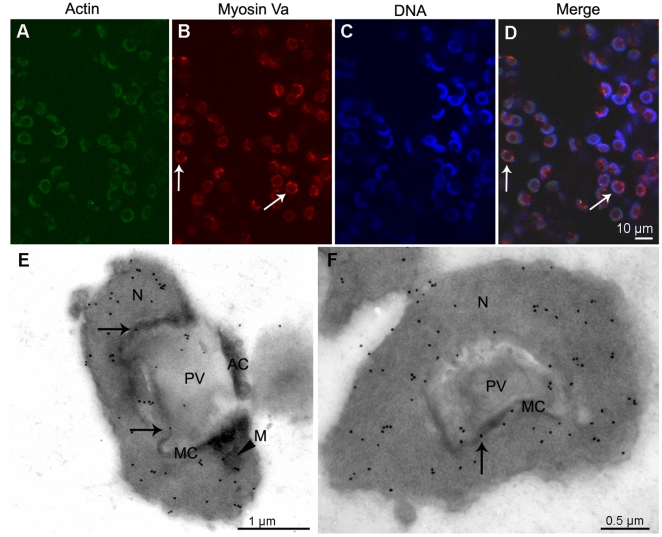
Localization of myosin Va at the beginning of late stage during *E. sinensis* spermiogenesis. (A–D) Immunofluorescent localization of myosin Va. Myosin Va concentrates in the nucleus (*arrows* in B and D). (E–F) Immunogold labeling of myosin Va. Prominent myosin Va labeling is present in the nucleus (N), and some associates with the nuclear membrane and the membrane complex (MC) (*arrows* in E, F), as well as mitochondria (M) (*arrowhead* in E). E. longitudinal section and F. cross section of the initiation of late stage.

**Figure 7:**
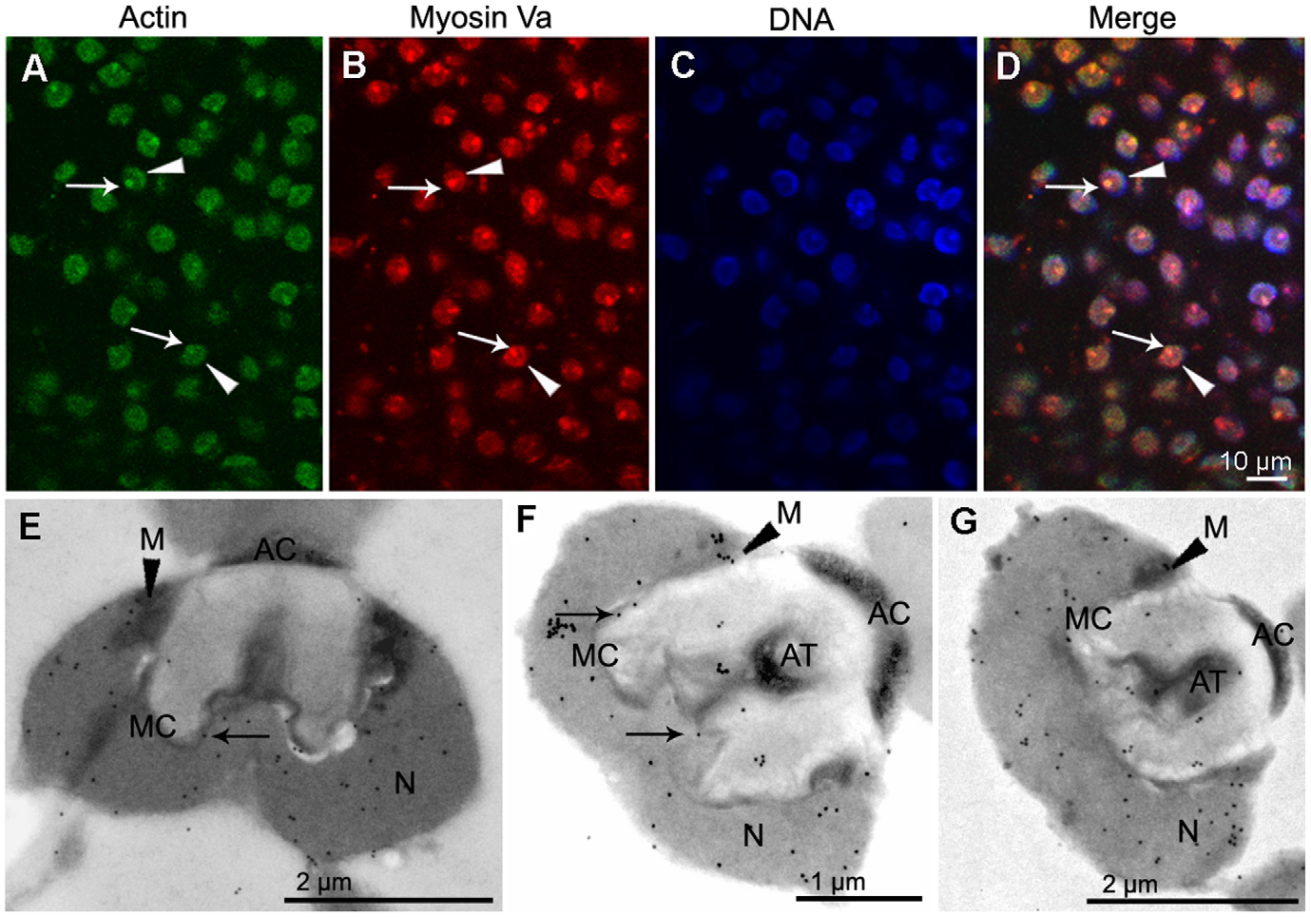
Localization of myosin Va at the late stage during *E. sinensis* spermiogenesis. (A–D) Immunofluorescent localization of myosin Va. Myosin Va distributes in the nucleus (N) (*arrowhead* in B,D) and acrosomal tubule (AT) (*arrow* in B,D), actin staining is present in the nucleus (N) (*arrowhead* in A,D) and acrosomal tubule (AT) (*arrow* in A,D), myosin Va colocalizes with actin (D). (E–G) Immunoelectron microscopy analyses of myosin Va. (E) When the acrosomal tubule (AT) begin to form, myosin Va associates with the nuclear membrane and the membrane complex (MC) (*arrow* in E). (F) After the acrosomal tubule (AT) formation, myosin Va bounds to the nuclear membrane and localizes in the membrane complex (MC) (*arrows* in F) and acrosomal tubule (AT). (G) Myosin Va localization in mature spermatozoon. Myosin Va distributes in the nucleus (N) and acrosomal tubule (AT). Mitochondria (M) are decorated with myosin Va labeling (*arrowheads* in E–G).

Immunofluorescent results displayed a distinct myosin Va distribution pattern at the late spermatid stage (Figure 6, 7). We discovered that at the beginning of the late spermatid stage, when the nucleus initiates to protrude at the bottom of the nuclear cup, accompanied by acrosome invagination, myosin Va allocates within the evaginated nucleus part and MC (Figure 6E, F). The mitochondria also display myosin Va immunoreactivity (Figure 6E). As spermiogenesis is in progress, myosin Va becomes associated with the evaginated nuclear membrane while the nucleus is protruding (Figure 7E). This is documented by the location of its immunofluorescence signal (Figure 7B, D). After the AT formation, a part of myosin Va is retained in the AT (Figure 7F). Immunofluorescent results demonstrated that AT displays a prominent actin staining which is colocalized with myosin Va (Figure 7D). In mature spermatozoon with well developed acrosome and nucleus, abundant myosin Va is distributed in the nucleus and AT (Figure 7G). The mitochondria are decorated with myosin Va at the late spermatid stage (Figure 7E–G).

## Discussion

**Figure 8:**
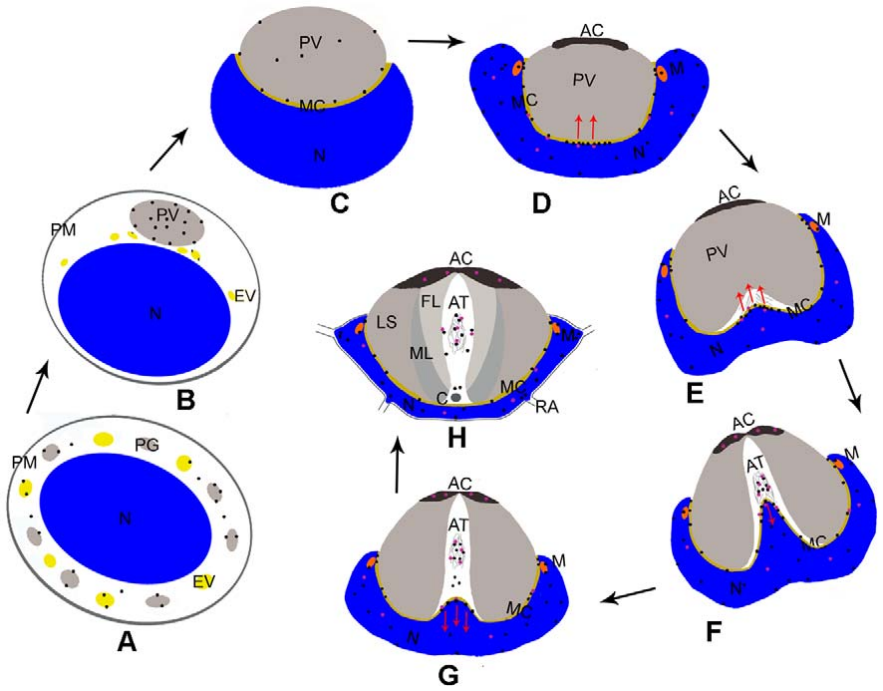
A model of myosin Va localization and function during *E. sinensis* spermiogenesis. The black point indicates myosin Va and the purple point indicates KIFC1. (A) In early stage, myosin Va is associate with the proacrosomal granule (PG) and the endoplasmic reticulum vesicle (EV) (*black point* myosin Va). (B, C) In mid stage, myosin Va localizes in the proacrosomal vesicle (PV) (B), as the nucleus (N) begin to wrap proacrosomal vesicle (PV), some myosin Va localizes to the membrane complex (MC) (C). (D–H) A hypothesis of the acrosomal tubule (AT) formation at late stage. At the initiation of late stage, myosin Va and KIFC1 exert a clutching force on the nuclear membrane at the bottom of the nuclear cup to push the membrane complex (MC) forward (red *arrows* in D, E); meanwhile, myosin Va at the other part of the membrane complex (MC) anchors proacrosomal vesicle (PV) to the nucleus and it also associates with the mitochondria (M) (D, E). When the acrosomal tubule (AT) reaches to the apical cap (AC), the evaginated nucleus (N) initiates to contract by pulling of myosin Va and KIFC1 (red *arrows* in F, G), some myosin Va interacts with actin in acrosomal tubule (AT), part of KIFC1 translocate to the apical cap (AC) (F, G). In mature spermatozoon, myosin Va localizes in the nucleus (N), membrane complex (MC) and acrosomal tubule (AT), while KIFC1 localizes in the apical cap (AC), nucleus (N) and acrosomal tubule (AT) (H).

Previous studies have proposed that myosin Va facilitates the transport of proacrosomal vesicles derived from the Golgi apparatus to the acrosome and anchors the developing acrosome to the acroplaxome plate during the nuclear elongation of spermatids in mammals [10], [40]. In addition, myosin Va is localized in the manchette and associated with manchette vesicles, indicating that it participates in the intramanchette transport [10], [41]. Myosin V was shown to be associated with the nucleus and to be involved in the investment cone formation and acrosome biogenesis during the *Drosophila* spermiogenesis [12]. During spermiogenesis in *E. sinensis*, PG and EV aggregate and then coalesce into PV adjacent to the nucleus. Later, the nucleus gradually invaginates to a cup-shape with about 20 radiating arms and surrounds the acrosome. Here we showed that myosin Va is associated with PG and EV at the early spermatid stage, and then decorates the PV membrane and MC situated between PV and the nucleus at the mid spermatid stage. Myosin Va is mainly distributed within the nucleus, part of which is associated with the evaginated nuclear membrane during AT formation. Myosin Va bounded mitochondria can be seen during the late spermatid stage. In mature spermatozoon, myosin Va is distributed in the nucleus and AT. A schematic diagram for myosin Va localization and AT formation during *E. sinensis* spermiogenesis is shown in Figure 8. Given the dynamic distribution and molecular characteristics of myosin Va during *E. sinensis* spermatogenesis, we propose that myosin Va plays an important role in spermatid differentiation.

### Myosin Va may be involved in PV biogenesis

In this study, myosin Va was found to be associated with the membrane of PG and EV in the early spermatids (Figure 4E, 8A). In the mid spermatids, PG and part of EV fuse to form PV, myosin Va signals were found on PV (Figure 5D, 8B). Subsequently the nucleus initiates to invaginate to wrap PV, and myosin Va is associated with the PV membrane and MC (Figure 5E, F, 8C). MC is situated between PV and the nucleus and contains F-actin and materials derived from degenerated EV and mitochondria, which may anchor PV to the nucleus. Myosin Va was demonstrated to associate with the ER in mouse melanoma cells and transport ER vesicles in squid axons [16], [42]. Myosin Va transports the proacrosomal vesicles generated by Golgi apparatus to the acrosome, and then connects acrosomal vesicle to the acroplaxome which is a F-actin containing plate [10], [40]. It is reasonable to assume that myosin Va has a role in transporting PG and EV to the acrosomal pole of the nucleus and facilitating them coalescing into PV, after which it also serves as a candidate protein to connect PV to the nucleus during nuclear invagination.

### Myosin Va may function in nuclear shaping and AT formation

The acroplaxome-myosin Va interaction is essential for acrosomal docking and nuclear shaping [10], [11], [40]. It has also been found to be associated with the nucleus during the spermatid individualization in *Drosophila* [12]. Acroframosome (AFS) consisting of F-actin and microtubule may serve as an anchor to tether the acrosome to the nucleus during spermiogenesis in caridean shrimp *Macrobrachium nipponense* [43]. MC contains F-actin and locates between the acrosome complex and the nucleus, which may have a similar role in anchoring the developing acrosome to the nucleus during spermiogenesis in Chinese mitten crab.

Du et al. (1988) have found that AT contains MC materials, however, the mechanism of AT biogenesis remains unknown [3]. KIFC1, microtubule-dependent motor protein, has been demonstrated to localize in the AC, AT and nucleus and may have a role in formation and maintaining of AC and AT structure during *E. sinensis* [9]. Nevertheless, no microtubule is existed in the AT [9], while AT is rich in actin (Figure 7A). Kinesin and myosin V have been demonstrated to be binding partners or act as tethers to function [33], [34], [36], [38]. Based on our result and the previous study, we propose a model of AT shaping (Figure 8D–H). Initially, the basal part of the cup-shaped nucleus protrudes to push the MC forward (Figure 8D, E). Myosin Va has a major distribution in the nucleus and resides in the nuclear membrane and MC (Figure 7E, 8E), therefore, it is possible that myosin Va and KIFC1 pulls the nucleus forward at the bottom of the nuclear cup; meanwhile, myosin Va in other parts of MC attaches the developing acrosome to the nucleus (Figure 8E). After AT reaches to AC at the tip of the acrosome, the nuclear convex contracts to the nuclear basal body via the drawing of myosin Va and KIFC1, while part of KIFC1 translocate to the AC which may function in the acrosome reaction (Figure 8F, G). A small amount of myosin Va and KIFC1 stay in AT interacting with F-actin (Figure 8H). It suggests that myosin Va and KIFC1 may work in concert to facilitate AT formation and fertilization.

Nuclear actin has been implicated in transcription and chromatin remodeling [44]–[46]. Myosin Va has also been proposed to function in nuclear compartmentalization and transcription [47]. What is even more interesting, in contrast to other types of spermatozoon, chromatin in the crab *Cancer pagurus* sperm is not compacted, which enable the nucleus to penetrate the oocyte more flexibly [48]. Combined with our result, it suggests that myosin Va and actin may participate in the nuclear organization of spermatids during spermiogenesis and fertilization.

In addition, we observed that mitochondria and MC which contained degenerated mitochondria display myosin Va labeling (Figure 5E, F, Figure 6E, 7 E–G, 8 C–H). Myo2, a Class V member, mediates mitochondrial motility directly in yeasts [18]. Therefore, myosin Va may be implicated in mitochondria localization and morphogenesis.

In summary, this work supports the notion that myosin Va is involved in the acrosome biogenesis. In addition, our result suggests a possible mechanism of AT formation. In this process, myosin Va and KIFC1 pull the nucleus to protrude, which is then pushing the MC forward to form the AT. After the AT reaches to the tip of acrosome, the evaginated nucleus retracts to the basal part of the nuclear cup by drawing of myosin Va and KIFC1. Furthermore, our work suggests a novel role of myosin Va in the nuclear morphogenesis during spermiogenesis of *E. sinensis*.
